# Draft Genome Sequencing of Three Glutaraldehyde-Tolerant Bacteria from Produced Water from Hydraulic Fracturing

**DOI:** 10.1128/mra.01232-21

**Published:** 2022-02-17

**Authors:** Stephen M. Techtmann, Andrew L. Baldwin, Dotun Aluko, Justin Andersen, Cole Becker, Grace Chandler, Steve Forgrave, Madelyn Jones, Ina Klasner, Jared Martini, Noah Mason, Ryleigh Parsons, Nick Peterson, Erik Reynolds, Lydia Schroeder

**Affiliations:** a Department of Biological Sciences, Michigan Technological University, Houghton, Michigan, USA; Montana State University

## Abstract

Here, we report the draft genome sequence of three glutaraldehyde-resistant isolates from produced water from hydraulic fracturing operations. The three strains were identified as *Marinobacter* sp. strain G11, *Halomonas* sp. strain G15, and *Bacillus* sp. strain G16. The genome sequences of these isolates will provide insights into biocide resistance in hydraulic fracturing operations.

## ANNOUNCEMENT

These three strains were isolated from produced water collected from hydraulic fracturing operations in the Permian basin of West Texas, USA. Hydraulic fracturing—also known as unconventional oil and gas production (UOG)—is the process of injecting water, sand, and other chemicals into oil -and gas-harboring shale formations (1). This process allows for recovery of oil and gas from previously difficult to access hydrocarbon basins. One of the chemical types commonly used in UOG operations is biocides (2). Biocides are antimicrobial chemicals used to control microbial growth in diverse settings. Glutaraldehyde is one of the most commonly used biocides in UOG operations (1, 2). There is concern about the potential for biocide use in UOG operations to lead to development of antimicrobial-resistant bacteria in the formation as well as in adjacent streams (3).

To better understand the diversity of biocide-resistant bacteria in the produced water, we isolated glutaraldehyde-tolerant strains of bacteria. Water samples were treated with 100 ppm of glutaraldehyde and incubated for 1 h. The samples were plated on nutrient agar and incubated at 30°C for 2 days. Colonies were picked and streaked three times to obtain isolates. The bacteria were routinely grown either in nutrient agar or nutrient broth. DNA was extracted using the Quick-DNA fungal/bacterial kits (Zymo Research). The 16S rRNA gene was sequenced using the primers 27F and 1492R to identify taxonomy (4). The resulting gene sequences were compared to the 16S rRNA sequence database at NCBI using blastn. Three isolates were selected for genomic analysis. The 16S rRNA gene from strains G11, G15, and G16 were all greater than 99% identical to Marinobacter vinifirmus strain FB1, Halomonas alimentaria strain YKJ-16, and Bacillus subtilis strain IAM 12118, respectively. These isolates were *Marinobacter* sp. strain G11, *Halomonas* sp. strain G15, and *Bacillus* sp. strain G16. Whole-genome libraries were constructed using the Nextera XT kit (Illumina) and sequenced on the Illumina MiSeq instrument using the MiSeq v3 600-cycle kit using a 2 × 300-bp paired-end sequencing run. All sequencing libraries were pooled and sequenced on the same MiSeq run.

Default parameters were used for all sequence analysis programs unless otherwise noted. Raw sequencing reads were quality controlled using Trimmomatic version 0.39 (5). Nextera adapters were trimmed from the reads. The leading and trailing 3 bp were trimmed, and the reads were quality filtered using sliding window trimming with a window size of 4 and quality cutoff phred score of 15. Any reads of less than 36 bp after quality filtering were removed. For G11, 412,903 paired reads survived trimming. For G15, 432,284 paired reads survived trimming. For G16, 816,767 reads survived trimming. The trimmed reads were then assembled using SPAdes version 3.15.3 (6) with kmer sizes of 21, 51, 71, 91, 111, and 127. The quality of the assembly was determined using QUAST version 5.0.2 (7). Annotation of the genomes was performed using Prokka version 1.14.6 (8). Table 1 details the statistics for the genome assemblies and annotation. Antibiotic resistance genes were annotated using the CARD database (9).

**TABLE 1:** Quality information and features of the *de novo* assembled strains

Feature	*Marinobacter* sp. G11	*Halomonas* sp. G15	*Bacillus* sp. G16
GC content (%)	57.9	65.6	43.9
Assembly length (bp)	4,040,679	3,811,422	4,258,983
Length of longest contig (bp)	288,494	318,971	411,992
No. of contigs	126	106	68
*N*_50_ (bp)	109,800	84,931	259,571
*L*_50_	12	15	7
No. of CDS^*a*^	3,791	3,538	4145
No. of rRNAs	8	6	13
No. of tRNAs	48	71	84

a CDS, coding DNA sequences.

The genome annotation revealed three resistance nodulation cell division (RND)-type efflux pumps in *Marinobacter* sp. G11 (two *adeF* and one *rsmA*). One RND-type efflux pump was identified in *Halomonas* sp. G15 (one *rsmA*). Two antibiotic inactivation genes and two RND-type efflux pumps were identified in *Bacillus* sp. G16. Previous studies have shown that the efflux pumps are important for resistance to glutaraldehyde (10). This finding suggests that efflux may be a mechanism allowing these bacteria to tolerate glutaraldehyde.

### Data availability.

The genome sequences are deposited at GenBank under accession numbers JAJSOZ000000000, JAJSPA000000000, and JAJSPB000000000, BioProject number PRJNA786782, BioSample numbers SAMN23721435, SAMN23721436, and SAMN23721437, and SRA accession numbers SRR17223254, SRR17223255, and SRR17223256.
